# Occupational Therapy Treatment Associated with Graded Motor Imagery (GMI) for the Recovery of Hand Function in Patients with Acquired Brain Injuries: Outcome Research

**DOI:** 10.3390/jcm14197060

**Published:** 2025-10-06

**Authors:** Francescaroberta Panuccio, Giovanni Galeoto, Angela Mastropierro, Giulia Marcellini, Andrea Marini Padovani, Anna Berardi

**Affiliations:** 1 Department of Human Neurosciences, Sapienza University of Rome, Viale dell’Università, 30, 00185 Rome, Italy; francescaroberta.panuccio@uniroma1.it (F.P.); giovanni.galeoto@uniroma1.it (G.G.); mastropierro.1908237@studenti.uniroma1.it (A.M.); andreamarinip@gmail.com (A.M.P.); 2 IRCCS Neuromed, Via Atinense, 18, 86077 Pozzilli, Italy; 3 Hospital San Giovanni Battista, 30, 00185 Rome, Italy; giuliamarcellini24@gmail.com

**Keywords:** rehabilitation, graded motor imagery, stroke, traumatic brain injury, occupational therapy

## Abstract

**Objective:** This study aims to evaluate the effectiveness of a combined rehabilitative program integrating Graded Motor Imagery (GMI) and Occupational Therapy in improving upper limb function and autonomy in individuals with acquired brain injuries (ABIs), including stroke and traumatic brain injury. **Methods:** Twelve patients (mean age of 56.4 years) underwent a six-week intervention combining GMI and Occupational Therapy. Outcome measures included the Disabilities of the Arm, Shoulder and Hand questionnaire (DASH), Jebsen Taylor Hand Function Test (JTHFT), Canadian Occupational Performance Measure (COPM), 12-Item Short Form Health Survey (SF-12), Numeric Rating Scale for pain (NRS), and Montreal Cognitive Assessment (MoCA). Assessments were conducted at baseline, post-treatment, and at 3- and 6-month follow-ups. Data were analyzed using the Wilcoxon signed-rank test. **Results:** Statistically significant improvements (*p* < 0.05) were found in upper limb function (DASH), occupational performance and satisfaction (COPM), and physical health status (SF-12 physical component). Specific gains in hand function—particularly in writing and eating—were detected using the JTHFT. No significant changes were observed in pain perception or mental health outcomes. **Conclusions:** The integration of GMI with Occupational Therapy appears to be a promising and well-tolerated intervention for enhancing motor function and daily life participation in individuals with ABI. Although the small sample limits generalizability, these preliminary findings support further investigation through larger, controlled studies.

## 1. Introduction

Acquired brain injuries (ABIs), defined as any damage to the brain that occurs after birth, are a leading cause of death and disability globally. They are usually divided into traumatic and non-traumatic brain injuries, which, respectively, include injuries caused by physical trauma or external impacts (e.g., motor vehicle accidents or falls) and conditions such as stroke, substance abuse, brain tumors, and brain hypoxia/anoxia [1]. Stroke, the most common form of non-traumatic ABI, is the third leading cause of death in higher-income countries and the leading cause of disability in adults, with approximately 15 million cases annually. The ischemic form is predominant, accounting for approximately 80% of cases [1,2]. Traumatic brain injury (TBI) represents another major global health challenge, being the leading cause of long-term trauma-related disability [3]. The global incidence is estimated at 50 million cases per year, with at least half of the world’s population experiencing a TBI episode in their lifetime [4]; survivors often experience impairments or disabilities that significantly reduce their quality of life [5].

An estimated 50 million people worldwide live with ABI-related disabilities, highlighting the need for preventive and rehabilitative interventions to reduce the impact on public health and improve patients’ quality of life [6]. Acquired brain lesions severely impair the use of the upper limb, affecting both motor and sensory functions due to damage to the sensory cortex, subcortical areas and the cerebellum [7]. Hand function can be significantly impaired, with loss of strength, dexterity, and motor changes such as spasticity, contractures, and abnormal flexion synergies. These effects result from neural and biomechanical changes, including chronic muscle denervation and excessive muscle coactivation [8]. These deficits significantly impair activities of daily living (ADLs), reducing autonomy and quality of life.

Graded Motor Imagery (GMI), developed by Moseley, is an innovative therapeutic technique that aims to “train the brain” to improve motor and sensory skills, manage pain and promote cortical reorganization [9]. It is based on the brain’s ability to reorganize itself through graded experiences, exploiting key principles of neuroplasticity. GMI is based on three key neurophysiological principles that explain how the brain can reorganize itself and recover motor functions: brain plasticity, pain-related cortical alteration and mirror neuron function. Neuroplasticity is the key mechanism that allows the brain to adapt, restoring altered motor patterns and improving general functionality [10]. Another important aspect concerns the changes in cortical mapping induced by chronic pain, significantly altering the cortical representation of the affected body areas, causing motor dysfunction and less precise movements [11]. Another fundamental concept underlying GMI is the function of mirror neurons, which are activated both during the execution of a movement and when observing someone else performing the same movement [12,13]. Mirror neurons play an essential role in activating mental representations of movements, facilitating motor recovery and integrating motor and sensory experiences [14].

GMI is based on a gradual protocol divided into three main phases, which stimulate specific brain areas, promoting motor recovery without triggering protective responses related to pain [9]. Each phase of the protocol is aimed at addressing specific aspects of motor recovery and pain perception and is composed of three specific elements provided in levels of increasing complexity in terms of time and difficulty, which are believed to reflect the gradual activation of cortical networks [15]. The three main phases of the GMI are: (1) implicit motor imagery (IMI), which consists of the ability to discriminate the right/left laterality of the specific body portion; (2) explicit motor imagery (EMI), which consists of imagining movements and postures of the affected body portion, creating a mental representation of the movement called “neurotag”; and (3) mirror therapy, a technique based on the use of a mirror on which the healthy limb is reflected, giving the patient the illusion of moving the affected limb [15,16].

The aim of the following study is to explore the application of the GMI protocol integrated with occupational therapy in the treatment of patients with ABI (stroke and traumatic brain injuries), with improving hand functionality, patient autonomy and quality of life. The hypothesis is that the integration of GMI with conventional Occupational Therapy will lead to clinically significant improvements in upper limb functionality, autonomy in daily activities, and quality of life in patients with ABI.

## 2. Methods

This quasi-experimental study [17] was conducted by the R.E.S. (Ricerca Evidenza e Sviluppo) research group from the “Sapienza” University of Rome; in the last few years, R.E.S. was involved in carrying out experimental studies, systematic reviews and validation of assessment tools on neurological and orthopedic rehabilitation [18,19,20,21,22,23,24]. This quasi-experimental study consisted of one hour of treatment per session, divided into 30 min of Occupational Therapy—focused on passive and active-assisted mobilization, gross and fine motor training, and sensory re-education of the affected upper limb—and 30 min dedicated to the gradual application of the three phases of the GMI protocol.

### 2.1. Study Sample

The patients selected for this study were recruited at the Department of Human Neurosciences “Policlinico Umberto I” and the San Giovanni Battista ACISMOM Hospital in Rome, from March 2024 to August 2024. To be eligible for inclusion, participants had to (1) be diagnosed with ischemic/hemorrhagic stroke or TBI; (2) be at least 18 years old; (3) have a score > 15 on the MOCA test for the level of cognitive function. Patients with global aphasia that prevented any form of communication, both verbal and non-verbal, visual, auditory and comprehension deficits, unilateral spatial neglect, and neurological disorders of other entities (e.g., multiple sclerosis, Parkinson’s disease) were excluded from the study. Before completing the enrollment, every patient has to read and sign an informed consent [25].

### 2.2. Experimental Treatment

Each intervention was structured with an initial assessment at baseline (t0) and a final evaluation at the end of the program (t1) to monitor changes over time. The GMI protocol followed the standardized three-phase sequence proposed by Moseley [16]: (1) Laterality Recognition, where patients were asked to identify the laterality (left or right) of hand images shown on flashcards depicting various positions, angles, and daily activities. (2) Explicit Motor Imagery, which involved imagining specific hand movements shown in the same flashcards, starting from simple gestures and advancing to complex daily tasks identified through the COPM. Patients were asked to mentally simulate movements in the first or third person, focusing on sensory experiences and pain perception. (3) Mirror Therapy, in which a sagittal mirror was placed on the table to reflect the unaffected limb, creating the illusion of normal movement in the affected limb. Exercises progressed from passive observation to synchronous active movements behind the mirror, aiming to restore motor function and sensorimotor integration through visual–proprioceptive mismatch.

The Occupational Therapy treatment included passive and active-assisted mobilization of the affected upper limb to maintain joint range and prevent complications, followed by activities to promote gross and fine motor skills [26,27]. Gross motor activities focused on strength and large movements, while fine motor tasks targeted dexterity, coordination, and object manipulation. Sensory re-education was also integrated, including tactile discrimination with textured materials, thermal-pain recognition, and proprioceptive exercises to enhance body awareness. This comprehensive approach aimed at improving upper limb functionality by addressing both motor and sensory components through a combination of traditional OT and GMI.

### 2.3. Outcome Measures

The Disability of the Arm, Shoulder and Hand (DASH). It is a self-administered questionnaire composed of 30 items aimed at evaluating the disability of the arm, shoulder, and hand of patients and understanding the level of interference in the QoL or activities of daily living (ADLs). It also provides two optional modules for work and sports/performing arts: the DASH-W (4 items) and the DASH-SM (4 items). The score goes from 0 to 5, where 5 is the worst for each question and 0 is the best. The arithmetic mean of the items is then transformed to obtain the final score from 0 to 100 for 30 items [28].

The Jebsen–Taylor Hand Function Test (JTHFT). This clinician-administered and performance-based test was developed to provide a standardized and objective evaluation of fine and gross motor hand function using simulated ADLs. It only assesses the speed, not the performance quality, so slower times reflect a less functional performance. The total score is the sum of time taken for each subtest: writing, turning over 3 by 5-inch cards, picking up small common objects, etc. Patients must perform all of the subtests with both the right and left hands, with the non-dominant hand tested first [29].

The Canadian Occupational Performance Measure (COPM). This is a semi-structured interview administered by the therapist that investigates self-care (activities of daily living, functional mobility), productivity (paid work or volunteer work, family management, school and play) and leisure (quiet, active leisure and social life). Once the problematic activities have been identified, the patient is asked to identify five problems in terms of importance. For each of them, performance and satisfaction are indicated by a score from 1 to 10. The higher the score, the better the performance and the greater the satisfaction [30].

The Numeric Rating Scale (NRS). This is a single-item question in which the patient must assess the subjective perception of pain rating it from 1 to 10, where 1 corresponds to “no pain” and 10 to “the worst pain imaginable”. The scale can be administered graphically or verbally [31].

The Montreal Cognitive Assessment (MOCA). It is a clinician-based test used to assess mild cognitive impairment across 7 cognitive domains: visuospatial/executive functions, naming, attention, language, abstraction, memory, and orientation. The maximum possible score is 30 [32].

### 2.4. Statistical Analysis

Statistical analysis was performed using IBM-SPSS version 23.00 Statistics for Windows software (version 23.0; IBM Corp., Armonk, NY, USA). Since the study sample is very small, results were analyzed using the median (± Standard Deviation) to avoid that possible abnormal or asymmetric values could influence the trend and give distorted conclusions. Considering the impossibility of ensuring a normal distribution of the data, the non-parametric Wilcoxon test was chosen, which does not require the assumption of normality and is more reliable for small samples. The Wilcoxon rank test was used to compare the median of the results between two groups and calculate a *p*-value, considering the results significant with *p* < 0.05. The median of the results was also associated with the standard deviation to highlight the range of dispersion of the data with respect to the median values.

## 3. Results

Between March and August 2024, 12 patients were recruited, 6 men and 6 women, with a mean age of 56.4 years (SD 16.51). The demographic characteristics of the sample are reported in Table 1.

### Data Analysis

The non-parametric Wilcoxon test for paired samples showed statistically significant results, as shown in Table 2.

## 4. Discussion

The aim of this study was to evaluate the effects of an integrated protocol of GMI and Occupational Therapy in post-stroke and TBI patients, observing any improvements in hand functionality, ADLs and quality of life (QoL).

Statistical analysis was performed using the non-parametric Wilcoxon test for paired samples, to compare the results at time t0 (pre-treatment) and t1 (post-treatment). The total score of the DASH scale showed a statistically significant improvement in upper limb functional abilities, specifically on the total score of the tool. This result suggests that this integrated protocol may have the potential to contribute to a concrete improvement in hand functionality, with a consequent positive influence on the performance of life activities. From a clinical point of view, these functional improvements can lead to a reduction in disability and a greater level of independence in daily activities [33]. However, the mean change did not reach the minimal clinically important difference (MCID) (10–15 points) [34], indicating that, while the results are statistically significant, their clinical impact should be interpreted with caution.

GMI, as highlighted by Moseley, is based on a progressive approach that includes motor imagery and body perception, and it is useful not only for motor recovery but also for the improvement of body image. Integration with occupational therapy seems to amplify these benefits, offering a holistic approach that addresses both the cognitive and physical aspects of rehabilitation [15,16]. No statistically significant results were obtained for the DASH-W and DASH-SM subscales, considered optional and designed to assess the impact of upper limb disability on work activities and sports/performing arts activities, respectively. In this study, not all patients were active in work or sports, thus reducing the number of responses for the two subscales, which may have influenced the statistical significance of the results.

The results obtained from the administration of the COPM indicate statistically significant improvements in the mean scores between t0 and t1, both in satisfaction and occupational performance; the increase in scores is particularly relevant from a clinical point of view since a greater satisfaction with occupational performance and ability can increase treatment engagement and long-term maintenance of progress [35]. The observed mean changes were greater than the established MCID of 2 points for both performance and satisfaction, reinforcing the clinical relevance of these results [36].

The same result is supported by the statistical significance between pre- and post-treatment scores of the SF-12 scale, which is also comparable with a randomized controlled trial conducted on 26 post-stroke subjects, although different measurement tools were used to assess QoL [37]. The mean increase in the SF-12 Physical Component exceeded the MCID threshold of 3–5 points, further supporting the clinical meaningfulness of the improvement [38]. However, there are few studies in the literature investigating the effects of GMI on QoL in post-stroke patients, but the articles available to date highlight a positive influence of GMI treatment on the quality of life of patients with phantom limb syndrome [39], CRPS [14] and painful diabetic neuropathy [40]. Instead, the lack of statistical significance in the scores of the mental health subscale (SF-SM) can be justified by the fact that occupational therapy interventions integrated with the GMI focuses mainly on the recovery of motor functionality and autonomy in ADLs; mental health support requires a targeted approach which has to be carried out by specialists in the psychological field, allowing patients to fully and adequately address their new emotional and psychological needs.

Analysis of NRS pain scores revealed no statistically significant reduction between t0 and t1. The lack of a significant reduction in pain may indicate that, although the combined treatment of GMI and Occupational Therapy led to improvements in functional ability and physical health (as demonstrated by COPM and SF-12 scores), it may not be sufficient to influence perceived pain in this kind of population. A 2013 review by Bowering et al. evaluated the effectiveness of GMI in patients with chronic pain [41], suggesting that the protocol seems to be more effective for peripheral neuropathic pain than for pain associated with central brain damage. Furthermore, variability in the sample may have introduced bias into the study. Including patients in the acute, subacute, and chronic phases of recovery could have further affected the results, as each phase is characterized by different features and requires distinct approaches to pain management [42].

The results obtained from the statistical analysis of the JHFT between the dominant and non-dominant hands revealed statistically significant results for the items relating to writing (ITEM 1) and feeding (ITEM 4) for the dominant limb, as well as for the item relating to lifting small objects (ITEM 6), with significant results for both the dominant and nondominant hands. The same analysis was performed comparing the scores for the affected and unaffected limbs; in this case, the analysis showed statistically significant results for the items relating to ITEM 4 and moving large empty cans (ITEM 6); overall, considering the statistical results for the dominant and nondominant hands, overall improvement in fine motor skills and manual dexterity was obtained. Within the study sample, 7 out of 12 participants were right-handed with left hemiparesis, meaning that their dominant limb was not affected at all. This aspect may also have influenced the results, as the patients did not show significant motor improvement, most likely because, despite the therapist’s supervision, they continued to rely entirely on the unaffected dominant hand for daily activities at home. Lateral dominance can contribute to reduced use of the affected limb, limiting the frequency and intensity of sensorimotor stimulation and repetitive practice—both essential for motor recovery [43]. The persistent reliance on the dominant, unaffected limb may therefore hinder functional improvement of the paretic side, as insufficient engagement of the impaired limb compromises the activation of neuroplastic processes crucial for motor relearning [44].

## 5. Limitations of the Study

The study was conducted as a preliminary pilot investigation, with the first limitation being the small sample size (*n* = 12), limiting the possibility of providing a generalizable statistical estimate. However, no a priori power analysis was performed, as the study was intended as a preliminary feasibility investigation of integrating GMI and OT [42,43,44]. In addition, variability among participants—such as differences in diagnosis, age, and severity of injury—may have influenced the observed treatment effects [45,46,47,48]. Since no subgroup analyses were performed, variability in diagnosis and clinical stage may have influenced the treatment effects. Increasing the sample size would allow for more accurate stratification based on demographic and clinical characteristics. Another important limitation is the absence of a control group, which limits the ability to attribute observed effects specifically to the intervention (e.g., spontaneous recovery or placebo effects).

It is also worth noting that, to date, no studies have specifically investigated the application of GMI in individuals with acquired brain injury. This lack of evidence limits the development of clear treatment guidelines and targeted intervention strategies. Future research should focus on larger cohorts and explore the effectiveness of GMI in well-defined clinical subgroups, considering the nature of the pathology and the phase of recovery (acute, subacute, or chronic), in order to optimize the therapeutic approach.

## 6. Conclusions

This outcome study suggests that the integration of a GMI protocol with Occupational Therapy may contribute to improvements in hand function, performance in activities of daily living, and perceived quality of life in individuals with ABI. These preliminary results support the relevance of further research involving larger samples and personalized treatment plans based on diagnosis and clinical phase. While the combined use of GMI and Occupational Therapy appears to be a promising approach, additional investigation is needed to better understand its impact and to address pain and psychological dimensions more effectively.

## Figures and Tables

**Table 1:** Demographic characteristics of the sample (*n*: 12).

		Frequency	Percentage
**Diagnosis**			
Stroke	Ischemic	3	25%
	Hemorrhagic	4	33.33%
	Cerebellar	1	8.33%
Traumatic Brain Injury		4	33.33%
**Gender**			
Men		6	50%
Women		6	50%
**Stage of the condition**			
Acute		3	25%
Sub-acute		5	41.67%
Chronic		4	33.33%
**Affected upper limb**			
Right		5	41.67%
Left		7	58.33%
**Handedness**			
Right		6	50%
Left		6	50%
**Job**			
Employed		9	75%
Retired		3	25%
**Hobby/Sport**			
Present		6	50%
Not present		6	50%
**Rehabilitative setting**			
Outpatients		4	33.33%
Intensive rehabilitation units		8	66.67%

**Table 2:** Wilcoxon test for paired samples pre-post test evaluation (*n*: 12).

Questionnaire	Pre-Treatment (t0)		Post-Treatment (t1)		Wilcoxon	*p*
	Mean ± SD	Median	Mean ± SD	Median		
DASH	63.3 ± 17.2	65	55.34 ± 17.6	52.5	−2.197	**0.028**
COPM Performance	3.31 ± 1.44	3.00	4.77 ± 1.56	4.40	78.00	0.002 *
COPM Satisfaction	4.22 ± 1.93	3.40	5.75 ± 1.91	5.60	75.50	0.004 *
SF-12 Physical Score	29.13 ± 8.86	25.02	34.85 ± 8.26	33.21	65.00	0.041 *
SF-12 Mental Score	41.83 ± 11.08	42.66	47.73 ± 11.32	47.13	60.00	0.099
NRS	5.58 ± 2.87	5.00	4.92 ± 2.71	5.50	13.5	0.28
JTHFT 1D	52.07 ± 37.61	35.57	47.48 ± 36.89	34.02	2.00	0.009 *
JTHFT 1ND	76.88 ± 36.28	64.35	75.69 ± 39.97	69.98	27.00	0.959
JTHFT 2D	34.49 ± 42.32	14.00	33.11 ± 45.98	10.42	17.00	0.155
JTHFT 2 ND	34.91 ± 39.06	15.88	30.00 ± 37.23	12.82	27.00	0.347
JTHFT 3D	34.00 ± 38.35	15.97	31.84 ± 35.33	15.45	18.00	0.182
JTHFT 3ND	33.08 ± 33.75	19.16	29.44 ± 28.43	15.73	26.00	0.308
JTHFT 4 D	38.89 ± 39.25	18.15	34.72 ± 35.35	15.82	6.00	0.016 *
JTHFT 4ND	49.76 ± 37.23	48.44	39.89 ± 29.99	38.67	13.00	0.075
JTHFT 5D	51.48 ± 65.42	15.43	38.35 ± 44.40	8.87	16.00	0.071
JTHFT 5ND	36.42 ± 40.90	16.55	40.76 ± 46.86	10.52	34.00	0.929
JTHFT 6 D	21.96 ± 34.	8.39	20.89 ± 34.32	7.15	2.00	0.006 *
JTHFT 6ND	20.52 ± 21.99	12.72	15.43 ± 14.89	8.51	7.00	0.012 *
JTHFT 7 D	28.18 ± 36.77	6.97	25.30 ± 37.14	6.97	15.00	0.110
JTHFT 7 ND	15.10 ± 15.14	8.76	18.26 ± 21.17	8.35	20.00	0.136
JTHFT 1A	78.34 ± 37.63	79.56	78.76 ± 42.88	69.98	23.00	0.935
JTHFT 2A	50.18 ± 49.17	19.97	45.70 ± 51.72	16.65	23.00	0.374
JTHFT 3A	47.74 ± 43.07	21.85	42.56 ± 38.31	21.12	21.00	0.286
JTHFT 4A	63.73 ± 44.00	55.93	51.58 ± 38.90	40.17	5.00	0.022 *
JTHFT 5A	67.45 ± 66.23	50.25	61.37 ± 51.53	62.84	27.00	0.594
JTHFT 6A	33.13 ± 36.11	18.65	28.24 ± 34.09	10.96	6.00	0.016 *
JTHFT 7A	28.87 ± 35.70	10.29	32.31 ± 37.41	11,20	24.00	0.424

DASH: Disability of the Arm, Shoulder and Hand; COPM: Canadian Occupational Performance Measure; SF-12: 12-Item Short Form Health Survey; NRS: Numeric Rating Scale; JTHFT: Jebsen-Taylor Hand Function Test; A: affected limb.* Statistically significant at *p* < 0.05.

## Data Availability

The authors confirm that the data supporting the findings of this study are available within the article.
